# The Role of the Hippo Pathway in Melanocytes and Melanoma

**DOI:** 10.3389/fonc.2013.00123

**Published:** 2013-05-16

**Authors:** Ji Eun Kim, Graeme J. Finlay, Bruce C. Baguley

**Affiliations:** ^1^Faculty of Medical and Health Sciences, Auckland Cancer Society Research Centre, The University of AucklandAuckland, New Zealand

**Keywords:** epidermal melanocytes, E-cadherin, cytoskeleton, merlin, cell proliferation

## Abstract

The Hippo signaling pathway comprises a series of cytoplasmic tumor suppressor proteins including Merlin and the Lats1/2 and MST1/2 kinases, and is thought to play a critical role in determining the sizes of organs and tissues. The Hippo pathway is regulated upstream by extracellular mechanosensory signaling arising from cell shape and polarity, as well as by a variety of extracellular signaling molecules. When active, the pathway maintains the transcriptional activators Yes-associated protein (YAP) and TAZ in phosphorylated forms in the cytoplasm, preventing cell proliferation. When the Hippo pathway is inactivated, YAP and TAZ are translocated to the nucleus and induce the expression of a variety of proteins concerned with entry into the cell division cycle, such as cyclin D1 and Fox M1, as well as the inhibition of apoptosis. The failure of the Hippo pathway has been implicated in the development of many different types of cancer but there is limited information available as to its involvement in melanoma. We hypothesize here firstly that the Hippo pathway is involved in maintaining density of cutaneous melanocytes on the basement membrane at the junction of the epidermis and the dermis, and secondly, that its function is disturbed in melanoma. We have analyzed a series of 23 low passage human melanoma lines as well as cultured normal melanoma, and find that melanocytes, as well as all melanoma cell lines examined express TAZ. Melanocytes and most melanoma lines also express YAP. E-cadherin, an upstream regulator of the Hippo pathway, and Axl, a receptor tyrosine kinase regulated by the Hippo pathway, are expressed in melanocytes and in several melanoma cell lines. These observations, together with published evidence for the presence of Merlin, Lats1/2, and MST1/2 in melanocytes and melanoma cells, support the hypothesis that the Hippo pathway is an important component of melanocyte and melanoma behavior.

## Introduction

The Hippo signaling pathway derives its name from the discovery of a set of four genes in *Drosophila* that together were found to control organ size. These genes specify a series of kinases and adaptor proteins including Hippo (Hpo), Warts (Wts), and Salvador (Sav), loss of function of which results in flies with enlarged, folded eyes and excess head cuticle, a “hippopotamus-like” phenotype (Wu et al., 2003). Subsequent studies have demonstrated an analogous pathway in humans, potentially providing an answer to the long-standing question in biology of how organ size is stabilized throughout life (Pan, 2012). In humans, mechanical signals mediated by cell–cell contacts and by interactions with the extracellular matrix generate signals which are integrated in space and time and form the heart of the Hippo pathway (Halder et al., 2012). Mechanical signals are complemented by those from membrane receptors including G-protein coupled receptors (GPCRs), which are known to respond to a number of ligands such as lysophosphatidic acid, sphingosine-1-phosphophate, glucagon, and epinephrine (Yu et al., 2011). Merlin, a product of the neurofibromatosis type 2 (NF2) gene (Li et al., 2012), is a key component of the pathway and associates with Kibra, a protein associated with memory performance (Xiao et al., 2011) and with Expanded, a tumor suppressor protein also associated with the Hippo pathway (Hamaratoglu et al., 2006). The Kibra–Merlin-Expanded protein complex leads to activation of the Hippo pathway by activating Mammalian Sterile 20-like kinase (Mst1/2), a homolog of Hippo in *Drosophila*, through autophosphorylation (Yu et al., 2010). Mst1/2, complexed with the scaffold protein Sav1 (the analog of Salvador), phosphorylates and activates the large tumor suppressor (Lats1/2) kinase, which is the homolog of Warts in *Drosophila* (Chan et al., 2005). Lats1/2 are also directly activated by the scaffold protein, Msp-one binder (Mob1) (Zhao et al., 2011). Kibra also contributes to the activation of Lats1/2 (Moleirinho et al., 2013).

In a number of cell types Lats1/2 kinase has been found to phosphorylate and inactivate the transcription co-activator Yes-associated protein (YAP), the homolog of Yorkie in *Drosophila*, as well as TAZ, the YAP paralog transcriptional co-activator with PDZ-binding motif (Hao et al., 2008). Phosphorylation of YAP at Ser127 by Lats1/2 generates a 14-3-3 binding site that leads to YAP cytoplasmic sequestration through 14-3-3 binding and consequent spatial separation from nuclear target transcription factors, preventing entry into the cell division cycle (Zhao et al., 2007). Furthermore, phosphorylation of YAP at Ser381, and of TAZ, leads to further changes and to ubiquitin-mediated proteasomal degradation (Zhao et al., 2010). Loss of phosphorylation allows YAP/TAZ to enter the nucleus and initiate a complex cascade of transcription events that lead to cell proliferation, cell migration, and suppression of anoikis, a form of apoptosis (Zhao et al., 2011). Merlin may also act in a fashion similar to that of β-catenin, translocating to the nucleus and stimulating transcription (Li et al., 2012). Expanded also directly associates with Yorkie in *Drosophila* to inhibit growth of the Hippo pathway by sequestering Yorkie in the cytoplasm (Badouel et al., 2009).

Studies of the Hippo pathway suggest it has a three-dimensional “sense” that is communicated across the whole organ and controls both cell proliferation and apoptosis. A possible example of such organ size control is provided by liver; it has been known for many years that surgical reduction of liver volume leads to extensive cell division and regeneration until the liver approaches its original size. The Hippo pathway has been hypothesized to have a role in this process (Avruch et al., 2011). In this review, we explore the hypothesis that the Hippo pathway is responsible for determining the overall number of cutaneous melanocytes and that changes in this pathway contribute to the development of melanoma.

## Melanocytes and Their Functions

Melanocytes are found in a number of locations including the eyes, ears, and brain but are particularly noted for their ability to form a two-dimensional network in skin at the junction of the dermis and the epidermis. Melanocytes are localized on the basement membrane, a layer of fibrous proteins which separates the dermis from the epidermis, and have a density of approximately 1000 cells per square millimeter (Gilchrest et al., 1979). This density is maintained throughout life. The skin comprises the epidermis, which contains melanocytes, keratinocytes, and Langerhans cells, and the dermis, which includes blood vessels, nerve cells, adipocytes, macrophages, and fibroblasts (Norris, 2011). All three epidermal cell types and many dermal cell types express toll-like receptors and contribute to recognition of pathogens in host immunity (Hari et al., 2010). Melanocytes on the basement membrane have the additional function of synthesizing melanin and transporting it in vesicles (melanosomes) to keratinocytes within the epidermis, thus protecting the epidermis from ultraviolet light (UV)-induced damage.

**Figure 1:**
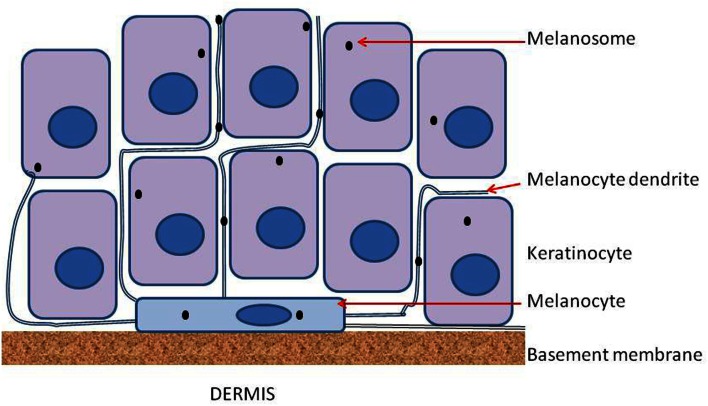
**Diagram of a melanocyte on the basement membrane and sandwiched between the epidermis and the dermis**. Melanocytes extend a number of dendrites into the epidermis and these serve to transfer melanosomes from each melanocyte to a number of keratinocytes. Connections between adjacent melanocytes also involve dendrites but are not shown in the figure.

**Figure 2:**
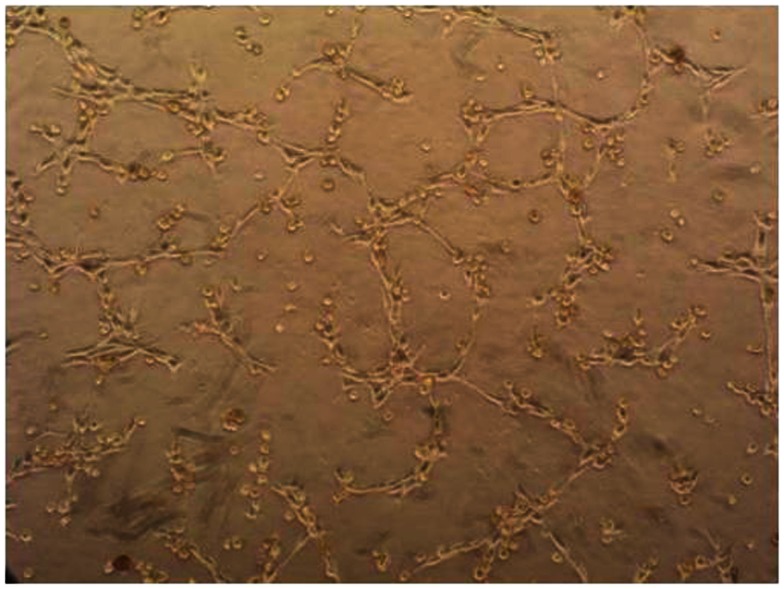
**Phase contrast photomicrograph of normal melanocytes growing on a layer of Matrigel**.

Epidermal melanocytes are strongly polarized and bind on one face to laminin molecules of the basement membrane via integrins including α3β1 and α6β1, and on the other face to other cells of the epidermis through long processes called dendrites (Fukunaga-Kalabis et al., 2006). Each melanocyte appears to interact with several dozen keratinocytes (Haass et al., 2005), as shown diagrammatically in Figure 1. Interestingly, when melanocytes are cultured on Matrigel, a mixture of proteins which approximates the basement membrane, they form a network of similar cell density, as shown in Figure 2. Both melanocytes and keratinocytes express E-cadherin and desmoglein, allowing the formation of adherens junctions between them and probably also between melanocytes in the network. The formation of dendrites, which may be controlled by Rac1 (Scott and Cassidy, 1998), allows contact to be made with multiple keratinocytes and the transport of melanosomes to the outer layers of the skin.

## Stimulation of Melanocyte Proliferation

Melanocytes, like fibroblasts, normally exist in a quiescent state but continuously preserve their ability to proliferate in response to cell loss or injury. The local production of reactive oxygen species (ROS) constitutes one of the main causes of cell injury and death (Fried and Arbiser, 2008). Melanin production is itself associated with free radical production (Arck et al., 2006) and environmental UVA and UVB are known both to generate ROS (Noonan and De Fabo, 2009) and to increase melanocyte density (Gilchrest et al., 1979). Inflammatory processes in the skin in response to pathogens also induce Langerhans cells and other epidermal cells to generate ROS. Replacement of melanocytes is an important facet of the maintenance of the epidermis and the induction of melanocyte proliferation may occur not only as a physiological response to cell injury or loss but also as a result of an oncogenic event such as an activating mutation. Examples of such events include activating mutations of the gene *BRAF* (Pollock et al., 2003), which activate the MEK/ERK pathway? and activating mutations of the gene *PIK3CA*, which activate the PI3K/AKT pathway (Hafner et al., 2007). An important cellular response to such oncogenic events is the induction of senescence. One potential cellular mechanism is provided by the p130-E2F4-DREAM complex; loss of this function leads to the activation of the protein p16, which mediates senescence (Hauser et al., 2012). This and other mechanisms are thought to contribute to the formation of moles or naevi, a collection of senescent cells within the skin epidermis.

## Hypothesis: The Hippo Pathway in Melanocytes

**Figure 3:**
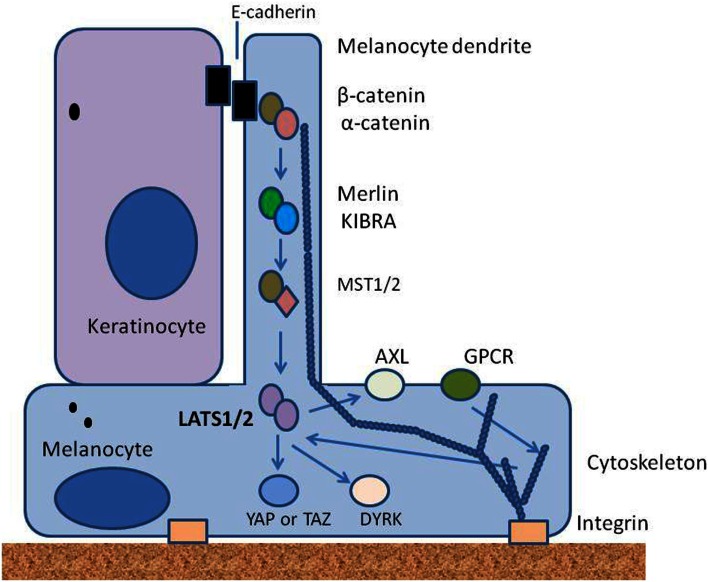
**Diagram showing some of the upstream elements of the proposed Hippo pathway**. Melanocytes are strongly polarized and receive signals on the one hand from the epidermis through adherens junctions with keratinocytes, and on the other hand from the basement membrane through integrins. They also receive signals through receptor tyrosine kinases such as Axl and through G-protein coupled receptors (GPCRs). All are likely to signal through kinases to Lats1/2; arrows indicate signaling events.

We hypothesize here that the overall density of melanocytes throughout life is controlled by elements of the Hippo signaling pathway. We suggest that mechanical signals mediated by contacts between melanocytes and the basement membrane, other melanocytes and keratinocytes are integrated in space and time to activate the Hippo pathway, as has been suggested for other tissues (Halder et al., 2012). Mechanical signals are complemented by biochemical signals, particularly from keratinocytes, and relayed from surface receptors, to cellular components through protein phosphorylation. Some of the proposed upstream elements of this hypothesis are shown in the simplified diagram in Figure 3. Melanocytes are maintained in a physically stretched state by interaction of integrins with elements on the basement membrane, as well as by the interaction of E-cadherin, desmoglein, and other adhesion molecules on dendrites with other cells, particularly adjacent keratinocytes; F-actin polymerization and depolymerization are thought to contribute to the extension and retraction of dendrites that interact with keratinocytes.

Melanocytes express E-cadherin, along with β-catenin and α-catenin (Larue et al., 2003), which by analogy with other cell types would be expected to interact with components of the cytoskeleton to form adherens junctions (Mareel et al., 1997; Shapiro and Weis, 2009). Merlin is known to be expressed by melanoma cells and may also be expressed by melanocytes; it is recruited to nascent adherens junctions and may signal through MST1/2 (Murray et al., 2012). Glutamate metabotropic receptors are GPCRs expressed by melanocytes (Hoogduijn et al., 2006) and may provide a link between glutamate, produced by keratinocytes, and Lats1/2 in an analogous fashion to that proposed for other GPCRs (Yu et al., 2011). Another potential link is the Axl receptor tyrosine kinase, which is located on melanocytes (Sensi et al., 2011), is activated by the expression of growth arrest-specific (GAS) factors produced by keratinocytes (Manzow et al., 1996) and is regulated by the YAP pathway (Xu et al., 2011).

**Figure 4:**
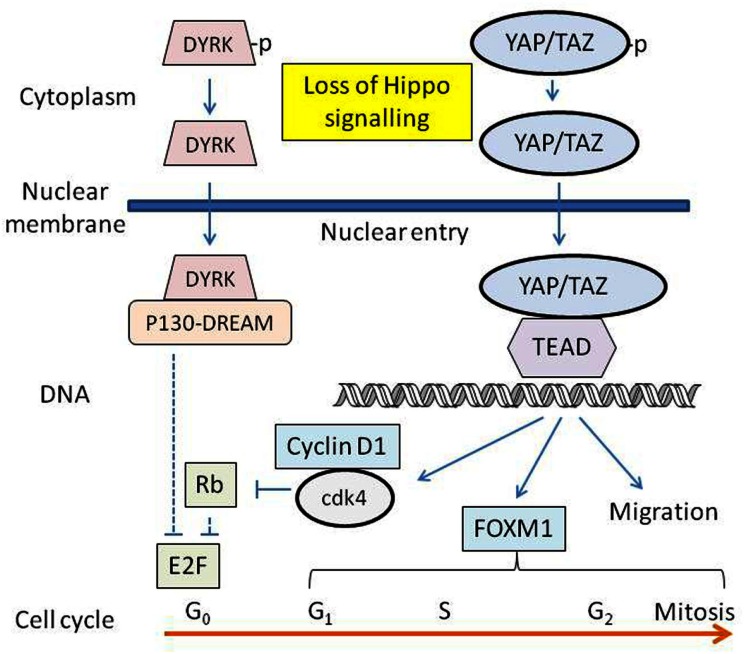
**Diagram showing some of the downstream elements of the proposed Hippo pathway**. The transcription factors YAP/TAZ-TEAD are activated, and DYRK-DREAM is inactivated by loss of Hippo signalling. Resulting transcription products are involved in regulating the synthesis of a number of proteins associated with cell proliferation and migration.

A scheme whereby YAP and TAZ participate in the control of proliferation of cultured melanocytes is shown in Figure 4. Signals for proliferation are provided by the culture substrate and the specific components of the growth medium, and it is possible *in vivo* that cell loss or injury results in loss of melanocyte contacts with the basement membrane and/or other cells, inhibiting LATS1/2 function and activating YAP/TAZ. Some of the downstream signaling pathways of activated YAP/TAZ are depicted in Figure 4. Dephosphorylated YAP/TAZ enters the nucleus, binding to and activating TEAD transcription factors, which in turn lead to increased transcription of target genes, increases in cell motility, invasion, anchorage-independent growth and proliferation, and resistance to apoptosis (Mizuno et al., 2012; Zhao et al., 2012). YAP-TEAD transcription induces *CCND1*, the gene encoding cyclin D1 (Cao et al., 2008) and *FOXM1*, a gene encoding a member of the Forkhead family of proteins (Mizuno et al., 2012). Cyclin D1 activates cyclin-dependent kinases 4 and 6 (cdk4 and cdk6), which in turn phosphorylate the retinoblastoma protein (Rb), allowing activation of E2F transcription factors. FOXM1 regulates the cdc25B protein phosphatase, cyclin B, polo-like kinase, aurora B kinase, and centromere proteins, controlling progression through S-phase and mitosis as well as cell cycle transitions from G1-phase to S-phase and from G2-phase to mitosis (Koo et al., 2012).

Lats1/2 also activates the dual-specificity tyrosine phosphorylation-regulated kinases (DYRK) (Tschop et al., 2011), which in turn phosphorylate and activate multi-protein complexes known as the p130-E2F4-DREAM (DP, retinoblastoma, E2F, MuvB) repressor complexes. These silence E2F target gene expression (Dick and Mymryk, 2011; Tschop et al., 2011). Dephosphorylated DYRK is no longer able to activate the p130-E2F4-DREAM complex, leading to derepression of transcription of genes under the control of activating members of the E2F family (Tschop et al., 2011), including cyclin E (Dick and Mymryk, 2011) and a number of proteins associated with DNA replication. Loss of Hippo activity might thus constitute a selective pressure for the inactivation of p16-mediated suppression of proliferation signaling and the emergence of melanoma cells. Several studies indicate the existence of cross-talk between the Hippo pathway and other signaling pathways. For instance, YAP activation has been shown to alter the function of the MAPK pathway (Kang et al., 2011). The Hippo pathway also inhibits the Wnt/β-catenin pathway by promoting interaction between TAZ and the Disheveled (DVL) protein of the Wnt pathway in the cytoplasm (Varelas et al., 2010; Azzolin et al., 2012), indicating a role for the Hippo pathway in morphogenetic signaling.

## Involvement of the Hippo Pathway in Melanoma

**Figure 5:**
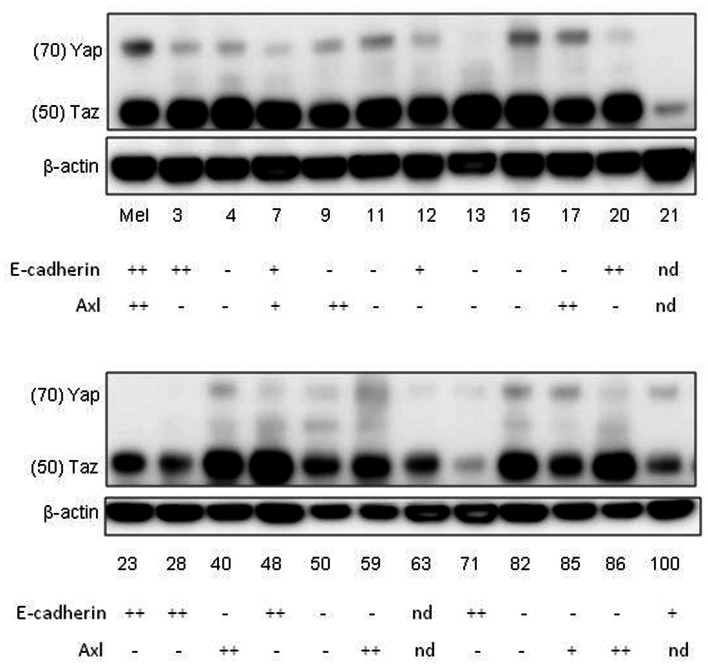
**Western blots of whole-cell extracts of cultures of normal melanocytes and of a number of melanoma lines, indicating expression of YAP and TAZ**. The numbers indicate the identities of members of the New Zealand melanoma collection (e.g., 3 = NZM3). Mel indicates data for normal melanocytes. Data for expression on western blots of E-cadherin and Axl, taken from another publication (Kim et al., submitted), are shown below the NZM numbers; – expression not detected; +weak expression; ++strong expression; nd not done.

To collect evidence for the involvement of the Hippo pathway in melanoma, a series of melanoma lines developed in this laboratory (Marshall et al., 1993, 1994; Kim et al., 2012) were analyzed for expression of some of the components and targets of the Hippo pathway (Figure 5). Many of the melanoma lines have been found to form networks on Matrigel (Zhao et al., 2005) in a manner similar to that of melanocytes (Figure 2), suggesting that they are capable of interaction with the extracellular matrix. Most of the lines were tested for expression of E-cadherin and N-cadherin (Kim et al., submitted), which are upstream elements of the Hippo pathway. The majority of lines had lost E-cadherin expression and replaced it with expression of N-cadherin, which is associated with a more invasive phenotype (Qi et al., 2005); a small proportion of cell lines expressed neither E-cadherin nor N-cadherin. All of the melanoma lines tested strongly expressed TAZ and many additionally expressed YAP. As shown in Figure 5, 35% of melanoma lines, as well as normal melanocytes, expressed Axl although this was not related to expression of cadherins. Another study reported that 38% of melanoma lines expressed Axl and postulated that expression was associated with motility and invasion (Sensi et al., 2011).

It is known that from animal models of melanoma that proliferation and invasiveness are promoted by YAP-TEAD (Lamar et al., 2012) and inhibited by Merlin (Murray et al., 2012). *CTGF* (Braig et al., 2011) and *GLI2* (Alexaki et al., 2010), two genes downstream of the YAP/TAZ-TEAD complex, have been associated with increased proliferation and invasiveness in melanoma. Taken together, these results support the involvement of YAP and TAZ in some stages of melanoma development. It is likely that the microenvironment of the melanoma may also be involved in YAP-TEAD regulation, for instance in the generation of ROS and cytokines. Further research needs to be carried out to characterize other elements of the Hippo pathway in melanoma, particularly upstream elements such as Lats1/2.

Our previous studies have suggested that two changes may be important to distinguish melanoma cell lines from cultured melanocytes: the partial loss of serum dependence of some intracellular signaling pathways (Kim et al., 2012) and the suppression of the p16 inhibitory pathway (Charters et al., 2011), which is common in melanoma (Hauser et al., 2012). Melanomas, as opposed to melanocytes, contain a number of mutated genes, raising the question of whether any of these mutations can affect the integrity of the Hippo pathway. Melanomas have a high frequency of mutant BRAF mutations, and in papillary thyroid cancer, expression of mutant BRAF is associated with inhibition of MST1/2 kinases (Lee et al., 2011); it would be interesting to determine whether this is also the case in melanoma. Moreover, BRAF mutations are associated with a Rac-dependent cadherin switch in melanoma (Monaghan-Benson and Burridge, 2013), suggesting a link to the cytoskeleton. A survey of mutant genes in melanoma revealed a high frequency of Rac1 mutations (Krauthammer et al., 2012); Rac1 acts to modify the cytoskeleton and loss could potentially change Hippo pathway regulation. The Ras association domain family gene *RASSF1* is frequently inactivated by promoter hypermethylation in a variety of human tumors including melanoma (Spugnardi et al., 2003; Richter et al., 2009) and its protein product RASSF is known to be a binding partner of MST1/2 kinases (Khokhlatchev et al., 2002), again suggesting a link to the Hippo pathway. The *GRIN2* gene, which codes for a subunit of the glutamate ionotropic receptor, is mutated in approximately 25% of melanomas (Wei et al., 2012). This receptor is involved in modulation of melanocyte dendrite morphology (Song et al., 2012) and might therefore also affect the Hippo pathway. Mutations in the *NF2* gene, which encodes Merlin (Figure 3) have been reported in a number of cancers including approximately 30% of melanomas and could have an important role in the efficacy of the Hippo pathway. Finally, FAT4 is known to inhibit cell growth by activation of the Hippo pathway and the *FAT4* gene is recurrently mutated in several types of human cancer including melanoma (Katoh, 2012).

## Conclusion

One of the fascinating features of the Hippo pathway is that it is able to integrate several different types of signaling, including those induced by cellular shape and adhesion changes, stress responses, and fluctuating concentrations of extracellular signaling molecules. It mediates cellular decisions on the control of proliferation, motility and cell death, and existing evidence indicates a complex and possibly redundant series of intracellular pathways (Halder et al., 2012). Melanomas are often thought of as developing mainly as the result of multiple genetic changes and a number of these may be linked to the function of the Hippo pathway. Furthermore, there is a strong possibility that extracellular signaling from the melanoma microenvironment may be important in tumor progression. The pathways underlying transduction of mechanical and cytoskeletal signals are now under intensive investigation and may provide a rich source of potential targets for the therapy of this disease.

## Conflict of Interest Statement

The authors declare that the research was conducted in the absence of any commercial or financial relationships that could be construed as a potential conflict of interest.
